# First pediatric electronic algorithm to stratify risk of penicillin allergy

**DOI:** 10.1186/s13223-020-00501-6

**Published:** 2020-12-04

**Authors:** Hannah Roberts, Lianne Soller, Karen Ng, Edmond S. Chan, Ashley Roberts, Kristopher Kang, Kyla J. Hildebrand, Tiffany Wong

**Affiliations:** 1 Division of Allergy and Immunology, Department of Medicine, Western University, St. Joseph’s Health Care, 268 Grosvenor St, London, ON N6A 3N3 Canada; 2 Division of Allergy and Immunology, Department of Pediatrics, University of British Columbia, Vancouver, Canada; 3 Division of Clinical Pharmacy, University of British Columbia, Vancouver, Canada; 4 Division of Infectious Diseases, Department of Pediatrics, University of British Columbia, Vancouver, Canada; 5 Division of General Pediatrics, University of British Columbia, Vancouver, Canada

**Keywords:** Amoxicillin, Antibiotics, Pediatrics, Penicillin, Allergy, Anaphylaxis, Infection

## Abstract

Beta-lactam allergy is reported in 5–10% of children in North America, but up to 94–97% of patients are deemed not allergic after allergist assessment. The utility of standardized skin testing for penicillin allergy in the pediatric population has been recently questioned. Oral drug challenges when appropriate, are preferred over skin testing, and can definitively rule out immediate, IgE-mediated drug allergy. To our knowledge, this is the only pediatric study to assess the reliability of a penicillin allergy stratification tool using a paper and electronic clinical algorithm. By using an electronic algorithm, we identified 61 patients (of 95 deemed not allergic by gold standard allergist decision) as low risk for penicillin allergy, with no false negatives and without the need for allergist assessment or skin testing. In this study, we demonstrate that an electronic algorithm can be used by various pediatric clinicians when evaluating possible penicillin allergy to reliably identify low risk patients. We identified the electronic algorithm was superior to the paper version, capturing an even higher percentage of low risk patients than the paper version. By developing an electronic algorithm to accurately assess penicillin allergy risk based on appropriate history, without the need for diagnostic testing or allergist assessment, we can empower non-allergist health care professionals to safely de-label low risk pediatric patients and assist in alleviating subspecialty wait times for penicillin allergy assessment.

## Main text

To the editor,

Beta-lactam allergy is reported in 5–10% of children in North America [1–3], but up to 94–97% of patients are deemed not allergic after allergist assessment [4–6]. Reasons for the disparity between perceived and true drug allergy include predictable antibiotic side effects and symptoms of underlying infection, frequently mistaken for adverse drug reaction. Recently, the utility of standardized skin testing for penicillin allergy in the pediatric population has been questioned as recent literature has demonstrated that penicillin skin testing in pediatric patients can be less predictive of risk of IgE-mediated allergy [7]. Oral drug challenges are the gold standard in definitively ruling out immediate, IgE-mediated drug allergy [6].

Given the high demand for allergist assessment for perceived penicillin allergy (which in most cases is erroneously labeled) [1–3], along with the lack of resources and allergists to assess these cases in a timely manner, we aimed to create a clinical algorithm which could be applied by a variety of health care providers, without the need for allergist assessment, to accurately identify low risk patients.

This study was approved by UBC C&W Research Ethics Board and informed patient consent was obtained. A structured penicillin allergy questionnaire was administered to the patient/parent by an antimicrobial stewardship pharmacist. The same questionnaire was administered separately by a pediatric allergist during their standard drug allergy consultation, and skin testing and/or drug challenge was conducted, as indicated. The allergist’s final assessment categorized the patient as (1) Allergic (IgE-mediated allergy or severe cutaneous/systemic adverse reaction) or (2) Not allergic and was deemed the “gold standard” decision.

All penicillin allergy questionnaires were independently reviewed by three pediatric allergists, a general pediatrician, a pediatric infectious disease specialist, and an antimicrobial stewardship pharmacist, hereafter referred to as the “assessors”. These assessors were blinded to the gold standard allergist decision. The assessors were asked to follow a clinical algorithm on paper and categorize patients into one of the following risk levels: (1) Possible IgE-mediated allergy, (2) Low risk for allergy, (3) Allergic (previously assessed by an allergist), or (4) Suspected severe cutaneous/systemic adverse reaction, based on the answers to the penicillin allergy questionnaires. The assessors then indicated an “action plan” for the patient. They chose either (1) Penicillin may be prescribed again or (2) Avoid penicillin and refer to pediatric allergist.

An electronic version of the paper clinical algorithm was created using Excel, which automatically calculated risk level and action plan based on the answers applied from the penicillin allergy questionnaires. The electronic version was developed to minimize human error, which could arise by applying the paper algorithm manually, and ensure each step is accounted for. Different versions of the algorithm were tested to determine which was the most accurate at classifying patient risk level, compared to the gold standard allergist diagnoses, without increasing the number of false negatives (Table 1).

**Table 1:** Assessment of penicillin allergy risk using an electronic algorithm, compared with the clinical algorithm and gold standard allergist diagnosis

	Gold standard allergist diagnosis		
	Allergic	Not allergic (n/%)	Total
Clinical Algorithm			
Allergic	9	58	67
Low risk	0	**37 (39%)**	37
Total	9	95	104
Initial Electronic Algorithm			
Allergic	9	52	61
Low risk	0	**43 (45%)**	43
Total	9	95	104
Electronic Algorithm (Permutation 2)			
Allergic	9	34	43
Low risk	0	**61 (64%)**	61
Total	9	95	104

Total number of patients included in the analysis: 104

Electronic Algorithm (Permutation 2): Sensitivity-9/9 = 100%; Specificity-61/95 = 64%

We performed a power calculation using a 0.05 alpha (standard) and 80% power to detect an effect size of 0.4. for a goal sample size of 204 assessments (104 total subjects). Descriptive statistics were compiled for all variables. The decisions for risk level were compared across all assessors and a kappa statistic was calculated. Comparisons were made between assessor decision of risk level and the gold standard allergist decision. The assessor decision of risk level was compared with the electronic algorithm, and the two different permutations of it. Sensitivity and specificity were calculated for each of the versions of the electronic algorithm, compared with the gold standard allergist decisions. Data analysis was performed using Stata 15.

From July 2016 to May 2018, 117 patients were approached and 107 participated in our study at BC Children’s Hospital (response rate = 91.5%). Due to missing information for 3 patients, 104 patients are included in the analysis. See Table 2 for patient information.

**Table 2:** Clinical and Demographic Patient Characteristics

Characteristic	Result
Participants	N = 104
Age at assessment, Median	5.59 (3.30, 9.45)
Male	57 (55%)
Female	48 (46%)
Symptom Onset for Adverse Drug Reaction	
≤ 2 h	35 (34%)
> 2 h	42 (40%)
Unknown	27 (26%)
Duration of symptoms	
≤ 48 h	43 (41%)
> 48 h	60 (58%)
Unknown	2 (2%)
Medical attention	
No medical attention	6 (6%)
Emergency room	33 (32%)
Family physician office	68 (65%)
Received beta-lactam antibiotic prior to reaction	35 (34%)
Received antibiotics since adverse drug reaction	15 (14%)
Personal atopic history	
Asthma	23 (22%)
Eczema	24 (23%)
Food Allergy	10 (10%)
Family history of atopy	71 (68%)

Of the gold standard allergist decisions, 91% (95/104) of patients were deemed “Not Allergic”. Eight patients on history were identified to have non-IgE-mediated, serum sickness-like reactions. Only one patient was labeled as having an immediate, IgE-mediated penicillin allergy, with a positive skin test, and was not challenged. Based on history alone, 37 patients (39%) were identified as low risk for penicillin allergy using the clinical algorithm, 43 (45%) using the initial electronic algorithm, and 61 (64%) with the final electronic algorithm (Table 1). A total of 71 patients deemed low risk for penicillin allergy pursued successful oral drug challenges without skin testing. This included all 37 patients identified as low-risk with the clinical algorithm and the 61 patients identified using the electronic algorithm. A total of 89 patients completed successful oral drug challenges with no immediate adverse reactions; the majority (80%) without skin testing prior to challenge. Only two patients developed delayed, mild skin rashes post challenge. One of these patients had mild symptoms of an upper respiratory tract infection at the time of challenge. She was re-challenged at a later date and the challenge was successful. The other child was re-challenged and developed a mild macular rash after one hour of observation without systemic symptoms.

When the assessors manually applied the clinical algorithm, 37 patients were low risk for penicillin allergy based on history alone (Table 1). In all cases where there was disagreement, the non-allergist had a more conservative decision, categorizing the patient as “Possible IgE-mediated allergy” with an action plan to refer to an allergist. The sensitivity of the clinical algorithm for allergy (based on the gold standard allergist diagnosis) was 100%.

**Fig. 1:**
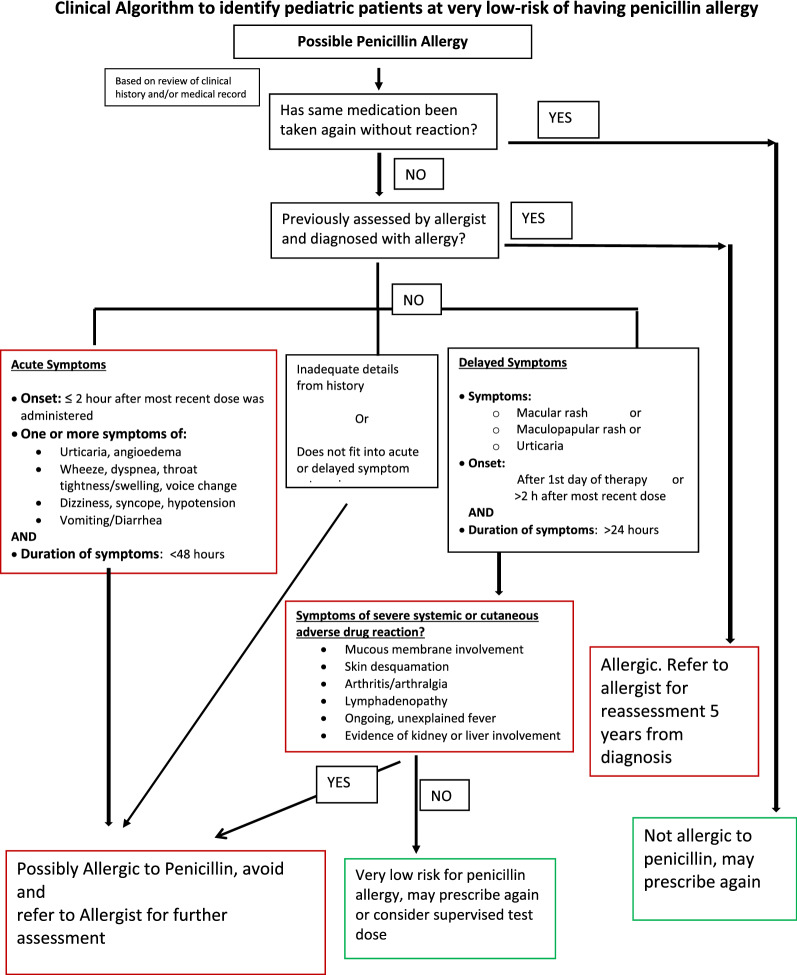
Clinical Algorithm to identify pediatric patients at very low-risk of having penicillin allergy

Applying the questionnaire answers into the initial electronic algorithm, 45% of patients were identified as low risk based on history alone. Altering the timing of resolution in the delayed symptoms category from > 48 h, to include symptoms lasting > 24 h (permutation 2), the number of patients identified as low risk increased to 64% (refer to Delayed Symptoms category in Fig. 1). In all cases the sensitivity of the electronic algorithm for allergy was 100%. This alternate version (permutation 2) of the algorithm had higher agreement with the gold standard allergist diagnosis (i.e. improved specificity) with no sacrifice in patient safety.

We demonstrated that by using a clinical algorithm (Fig. 1), a variety of pediatric health care professionals were able to safely and accurately risk stratify penicillin allergy. The electronic version of the algorithm further minimizes the risk of human error and captured an even higher percentage of low risk penicillin allergy patients than the paper version, making it superior. Although the paper and electronic algorithms are the same, and technically the paper version should yield the same result as the electronic version if users were to apply them correctly every time, they did not yield the same results. The most likely cause is that physicians were not following the algorithm on paper precisely and instead have personal bias related to past experiences and differences in personal risk threshold, that affect the final decision. Additional studies using this algorithm on an even larger scale in outpatient settings will be important to further corroborate our results and confirm safety.

A limitation of our study is that our population comes from only one pediatric center. In addition, our recommended algorithm relies on recall of the possible reaction, which may not be accurately reported for those with remote histories. Fortunately, remote histories are less likely in the pediatric population compared to the adult population. It is worth noting that timing of resolution of symptoms can depend on treatment. Reassuringly, even if symptom duration is decreased due to medication administration, the worst case scenario typically leads to a patient being placed in the high risk category, which does not reduce safety of the algorithm/patient, only potential delay in de-labeling while awaiting allergist consultation. A detailed clinical history, including specific management taken, is important in drug allergy assessment to ensure recognition of severe, delayed reactions which are contraindications to oral challenges. Six eligible low risk patients did not complete oral drug challenges secondary to being lost to follow-up or patient preference. We suspect our numbers for successful challenges would have been even higher with increased patient participation.

To our knowledge, this is the only pediatric study to assess the reliability of a penicillin allergy risk stratification tool, using both paper and electronic clinical algorithms. By using an electronic algorithm, we identified 61 patients (of the 95 deemed not allergic by gold standard allergist decision) as low risk, with no false negatives and without the need for allergist assessment or skin testing. Consistent with previous studies, our results confirm that the majority of patients with suspected penicillin allergy do not have immediate, IgE-mediated drug allergy [6, 8–11].

We demonstrate in this study that an electronic algorithm can be used by various pediatric clinicians when evaluating possible penicillin allergy to reliably identify low risk patients. We identified the electronic algorithm was superior to the paper version, capturing an even higher percentage of low risk patients. Electronic algorithms have an advantage over manual/paper algorithms in that they can be converted into a webpage or an application that can be easily accessed by clinicians using electronic devices, which is advantageous. They additionally have the ability to be incorporated into electronic health records.

By creating an electronic algorithm to accurately assess penicillin allergy risk based on appropriate history, without the need for diagnostic testing or allergist assessment, we can empower non-allergist health care professionals to identify and safely de-label low risk pediatric patients. This will assist in alleviating subspecialty wait times for penicillin allergy assessment, by ensuring that referrals to allergists are reserved for children with a higher probability of true, IgE-mediated drug allergy or severe, delayed reactions.

## Data Availability

Not applicable.

## Abbreviations

IgE: Immunoglobulin E
